# Comparative genomics reveals a widespread distribution of an exopolysaccharide biosynthesis gene cluster among Vibrionaceae

**DOI:** 10.1186/s13104-018-3214-z

**Published:** 2018-02-06

**Authors:** Lou Lebellenger, Véronique Verrez-Bagnis, Delphine Passerini, Christine Delbarre-Ladrat

**Affiliations:** Ifremer, Atlantique Center, Biotechnology and Marine Resources Unit, Marine Ecosystems and Marine Molecules for the Biotechnologies Laboratory, Rue de l’Ile d’Yeu, BP21105, 44311 Nantes Cedex 3, France

**Keywords:** Vibrionaceae, Exopolysaccharide, Genetic biodiversity, Biosynthesis, *Vibrio diabolicus*, HE800 EPS

## Abstract

**Objectives:**

The *eps* locus in *Vibrio diabolicus* is involved in the production of the biotechnologically valuable HE800 EPS. In this study, the distribution and diversity of similar *eps* gene clusters across Vibrionaceae and its variability in relation to phylogenetic relationship were investigated. The aim was to provide a better knowledge of the *eps* gene cluster importance and to facilitate discovery of new EPS with potent interesting bioactivities.

**Results:**

Seventy percent of the 103 genome sequences examined display such an *eps* locus with a high level of synteny. However, genetic divergence was found inside some monophyletic clades or even between some strains of the same species. It includes gene insertions, truncations, and deletions. Comparative analysis also reveals some variations in glycosyltransferase and export systems genes. Phylogenetic analysis of the Vibrionaceae *eps* gene clusters within Vibrionaceae suggests a vertical transfer by speciation but also pinpoints rearrangement events independent of the speciation.

**Electronic supplementary material:**

The online version of this article (10.1186/s13104-018-3214-z) contains supplementary material, which is available to authorized users.

## Introduction

**Fig. 1:**
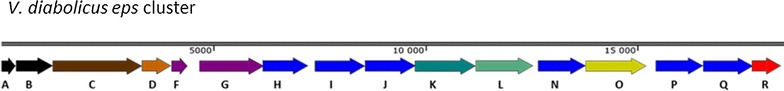
Genomic structure of *eps* cluster of *V. diabolicus*. Scale: base pairs. Genes are labeled after the *Aliivibrio fischeri* orthologous genes [16]. Encoded proteins are: A: anti-sigma factor antagonist; B: regulatory protein; C: export protein (Wza); D: chain length regulator (Wzc); F: sensor kinase; G: response regulator, transcriptional activator; H, I, J, N, P, and Q: glycosyltransferases; K: flippase (Wzx); L: polymerase (Wzy); O: chain length determinant (Wzz domain); R: undecaprenyl phosphate sugar phosphotransferase (priming GT)

The animal glycosaminoglycans are glycopolymers with key roles in cell physiology and pathologies [1]. The marine bacterium *Vibrio diabolicus* synthesizes the HE800 EPS, which shares some structure and bioactivity features with the glycosaminoglycan hyaluronan (HA) [2–4]. Thanks to its visco-elastic properties and biological properties on the cartilage and skin, HA is used in osteoarthritis treatment, ophthalmology, wound healing and in cosmetics. HE800 EPS has demonstrated its efficiency in bone and skin regeneration [5–7]. Its biosynthesis *eps* gene cluster has been identified (Fig. 1) [8] and appears as a good model to investigate *eps* loci in marine bacteria and find innovative glycosaminoglycan-mimetics for human health.

In this study, we investigated the distribution of orthologous *eps* gene clusters across Vibrionaceae and gene variations in relation to phylogenetic relationship.

## Main text

### Methods

#### Assembly collection

One hundred and three publicly available genome sequences of Vibrionaceae members, that cover several clades, were selected (Additional file 1).

Assemblies were obtained from NCBI [9] and three files were used: rna_from_genomic.fna for 16rDNA based phylogeny, protein.faa for homology search and cds_from_genomic.fna for genomic context evaluation.

#### Identification of the eps orthologous clusters

Proteins orthologous to *V. diabolicus* HE800 biosynthetic cluster were searched in the downloaded genomes by blastp comparisons using the standalone BLAST+2.2.30 package [10]. A local database gathering the 103 genomes was formatted with the makeblastdb program; a multifasta file containing the 16 *V. diabolicus eps* genes was used as the blastp query.

Alignment length ratios were calculated as follows: MinLrap = Lmatch/min(Lprot1, Lprot2) and MaxLrap = Lmatch/max(Lprot1, Lprot2) where Lmatch = match length, Lprot1 (or Lprot2) = protein 1 (or 2) length and min (or max) = minimum (or maximum) of the two values [11]. These values indicate, when both are equal to 1, that both whole proteins align. Alignment length ratios (close to 1), similar protein sizes, low expectation value (< 10^−25^) and identity percentage (above 40%) were taken into account to identify the first protein (usually A); genomic context was further inspected to evaluate if neighboring genes encode proteins which also share homology with *V. diabolicus* cluster.

#### In silico analyses of the protein coding sequence sets

Proteins encoded by the genes inserted between D and F were identified by Blast search against the NCBI database [12]. A phylogenetic tree was constructed using a concatenate of proteins; sequences were aligned with COBALT (https://www.ncbi.nlm.nih.gov/tools/cobalt/) and phylogenetic analyses were conducted with MEGA software v7 using the Neighbor-Joining method [13, 14].

#### Multilocus sequence analysis (MLSA)

Five housekeeping genes were used for strains MLSA phylogeny [15] (Additional file 2). Genes were aligned with MAFFT version 7. Because not all gene sequences are full length, positions 1–562, 227–893, 391–892, 442–1027, and 448–1073 (*V. diabolicus* numbering) of *pyrH*, *gapA*, *mreB*, *gyrB*, and *topA* genes, respectively, were concatenated. Phylogenetic tree was constructed with MEGA 7 using the Neighbor-Joining method [13, 14].

### Results and discussion

#### Distribution of the eps gene cluster in Vibrionaceae

**Fig. 2:**
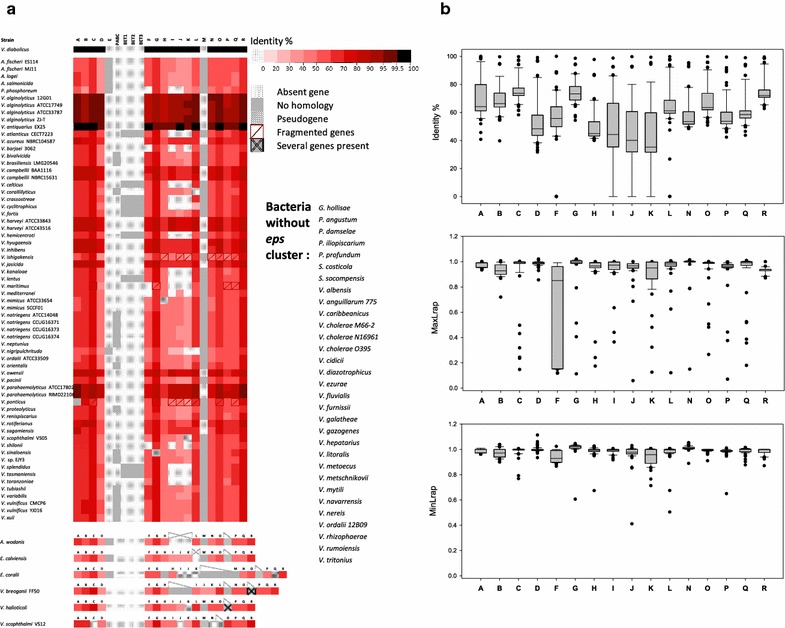
Biodiversity of the *eps* cluster among Vibrionaceae. **a** Protein components of the orthologous clusters with the identity % to *V. diabolicus* protein. Color legend is located at the top right portion, red level shows increasing identity by from light red to black. Dotted position are absent proteins, grey ones are proteins that do not exist in the HE800 cluster. **b** Boxplots showing distribution of data set from identity %, MaxLrap and MinLrap of A, B, C, D, F, G, H, I, J, K, L, N, O, P, Q and R proteins in comparison with *V. diabolicus* proteins. In the boxes, the 25th, 50th and 75th percentiles are indicated by the bottom, middle and top lines respectively. Whiskers show the 10th and 90th percentiles. Individual dots are the outliers. Absent genes were withdrawn from the min and maxLrap diagrams

Seventy-two orthologous *eps* clusters were discovered, while none could be found in the remaining 31 genomes, which include *Grimontia* and *Salinivibrio* members (Fig. 2a, Additional files 3, 4). *V. ichthyoenteri* was found to possess an *eps* gene cluster but it was not further analyzed because the cluster is split at the ends of two distinct contigs.

#### Variability of the eps gene cluster

The organization of the gene clusters was highly conserved, particularly in two syntenic blocks: the 5′ end of the cluster (A, B and C genes) and the 3′ end which always encompasses gene R. The species possessing the *eps* cluster the most similar to the *V. diabolicus* one, are *V*. *antiquarius* Ex25, *V*. *alginolyticus* and *V*. *parahaemolyticus* (Fig. 2a). The most conserved proteins are A, B, C and G, which are involved in regulation, O which is responsible for chain length regulation and R, which initiates polymerization; they shared more than 70% identity with *V. diabolicus* orthologs (Fig. 2b). The A protein is an anti-sigma factor antagonist and a key regulator of biofilm formation; the deletion of A gene in *V. fischeri* (*sypA*) inhibits biofilm formation and thus squid colonization [16]. The good conservation of both B and C proteins suggests they are important for the EPS biosynthesis, although their functions remain undetermined. D protein which functions as a chain length regulator [17] is conserved; an identity of about 50% with that encoded by *V. diabolicus eps* cluster suggests that its activity can be variable (Fig. 2b, Additional file 3).

The G gene, that codes for a σ54-dependent activator of *syp* locus transcription in *A. fischeri,* is also conserved [18–22] (Fig. 2b, Additional file 3). F, the other regulatory protein, is a hybrid sensor kinase which phosphorylates SypE and SypG in order to regulate biofilm formation in *V. fischeri* [20, 23]. F protein seems to be particularly subjected to variation (Fig. 2b), as it appears truncated in *V. diabolicus eps* cluster, whereas some other species carry a full copy [8, 17].

The *V. diabolicus eps* cluster encompasses 6 glycosyltransferases (GT), which biosynthesize the EPS repeating unit, and one priming GT (R), which initiates the EPS biosynthesis. Among the considered Vibrionaceae strains, 16 are lacking at least one of the *V. diabolicus* GTs. The most often observed difference is the absence of both I and J in 12 *eps* clusters (Fig. 2a). I protein has only a weak role in the EPS production, adding a branch on the polysaccharide backbone late in the biosynthesis process [17]. In addition, several GT genes are fragmented or annotated as pseudogenes. In *V. breoganii* FF50 and *V. halioticoli,* the P, Q and R gene group is separated from the rest of the *eps* cluster by, respectively, a 60 kb-long and a 5 kb-long sequence. Therefore, these genes may not be transcribed together with other *eps* genes. However, the PQR segment might be sufficient to synthesize some oligosaccharides, as in *A. fischeri*, the *syp PQR* segment constitutes an operon [24]. When present, H, I and J proteins display about 50% identity with the corresponding *V. diabolicus* ones. N, P and Q are slightly more conserved (about 60% identity) suggesting only slight putative differences in substrate and acceptor specificities. P, Q and R proteins exhibit an overall very high conservation across Vibrionaceae. They have been hypothesized to be related to the repeating unit biosynthesis [8], which could thus likely be predicted to be rather similar in Vibrionaceae EPS.

The HE800 EPS export system involves the periplasmic protein Wza (C protein), the oligosaccharide translocase Wzx (K protein), the polymerase Wzy (L protein) and the putative polysaccharide biosynthesis chain length regulator Wzc (O protein). It has been previously established that K protein is essential for EPS repeating unit translocation across the inner membrane [8, 17]. But 15 of the *Vibrionaceae* studied species are devoid of this protein, suggesting that they are not able to produce, or at least export, an EPS molecule [17]. L gene is absent in *Enterovibrio calviensis*, and classified as pseudogene in *V. coralliilyticus*. Both genes K and L are absent in *V. nigripulchritudo* (Fig. 2a). In all these strains, if the biosynthesis of the repeating unit occurs, it may accumulate most likely in the cytosol. Indeed, the proteins encoded by their *eps* cluster do not enable the repeating unit to be translocated across the membrane nor polymerized [8, 25].

Between D and F genes, additional individual genes or a group of three genes were found in several of the studied Vibrionaceae (Fig. 2a). One of these proteins (E) is homologous to *A. fischeri* SypE which is a two-component response regulator protein inhibiting SypG-mediated phenotypes and biofilm formation [19]. Another individual gene codes for a periplasmic component of an ABC type phosphate/phosphonate transport system (PABC). Phosphonates can be found as side groups on several macromolecules including polysaccharides [26]. On the other hand, EPS are known to form a slime around cells to sequester several compounds and could therefore be involved in bioremediation. But it is not clear why only the PABC periplasmic component is present while some Gram-negative bacteria possess a full copy of the phosphonate biosynthetic operon beside EPS biosynthesis genes [26]. For eight of the strains studied, three genes, coding for a glycine betaine/l-proline ABC transporter substrate-binding protein, a permease and an ATP-binding protein, are located between the genes D and F. As these proteins are homologous to the ProVWX components of the ProU transporter in *Escherichia coli* K12 [27], they may contribute to the uptake of glycine betaine which participates in bacterial osmoregulation, cryoprotection and protection against desiccation [28]. Betaine containing molecules can also constitute a source of phosphorus [29]. Several polysaccharides, especially the anionic ones, have been described to interact with glycine betaine compounds [30]. The presence of such transporter within EPS biosynthetic clusters could suggest adaptation of the bacterial strain to specific environmental conditions.

*Enterovibrio coralii* carries a glutamine-fructose-6-phosphate aminotransferase between H and N genes. It catalyzes the rate-limiting step in the synthesis of UDP-*N*-acetylglucosamine [31, 32] which is a precursor for both polysaccharide synthesis and cell growth in *E. coli* [33].

Several transposase and integrase genes have been identified in *V. breoganii* and in *V. scophthalmi* VS-12. These proteins allow insertion of mobile elements and thus recombination events [34, 35].

The *syp* locus of *A. fischeri* encodes the additional SypM, an *O*-acetyltransferase [17]. It has also been identified in 52 strains over the 103 studied ones (M, Fig. 2a). This could suggest the presence of *O*-acetyl groups in the final putative molecule.

#### Phylogenetic relationships

**Fig. 3:**
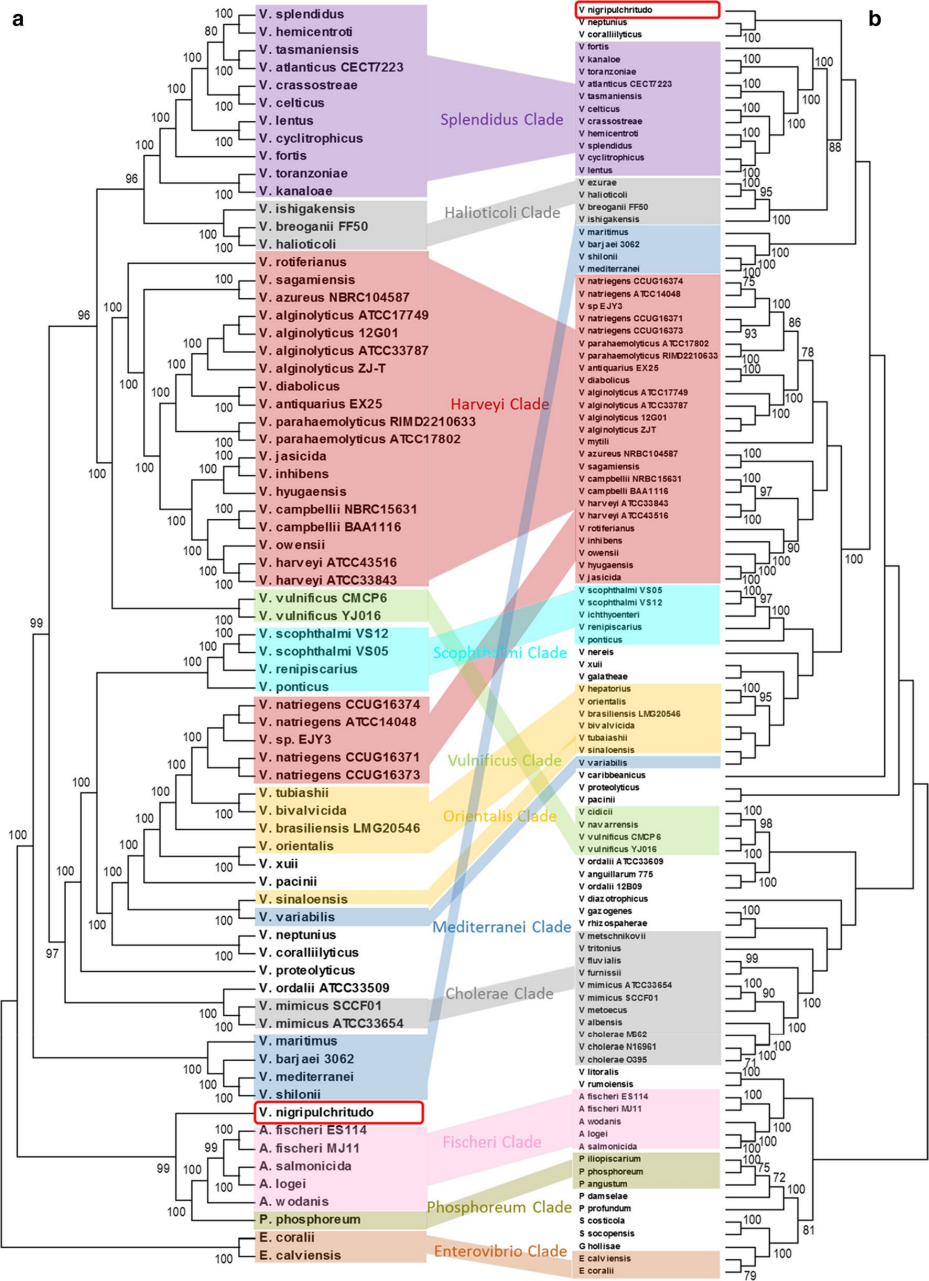
Evolutionary relationships of concatenated proteins encoded by *eps* gene clusters (**a**) and MLSA phylogenetic relationships between strains (**b**). Main monophyletic clades are also indicated in both trees. Concatenated proteins were aligned with COBALT and MLSA five-gene dataset was aligned with MAFFT. Phylogenetic trees were constructed with MEGA version7 using the Neighbor-Joining method [13, 14] with the Poisson correction method for proteins [38] and the Jukes Cantor substitution model for MLSA [39]. Bootstrap values (1000 replicates) are shown next to the branches when higher than 70 [40]

The MLSA phylogenetic tree (Fig. 3b) shows congruence in the clustering of the large majority of strains with trees previously described [15]. Concatenated proteins are generally clustered in the monophyletic clades (Fig. 3a). However some exceptions are noticed. *V. natriegens* species and *V.* sp. EJY3 are the sole strains of the Harveyi clade which possess the PABC protein. PABC protein was found in 17 strains belonging to 6 different clades (Harveyi, Orientalis, Nereis, Mediterranei, Coralliilyticus and Vulnificus); these clades also encompass other members possessing *eps* cluster that does not encode this protein. *V. mytili* is the only Harveyi clade member (over 25) that does not share the *eps* cluster. The concatenated proteins of *V. nigripulchritudo* (Nigripulchritudo clade) appear isolated as they miss K and L proteins. *V. nigripulchritudo* is also the sole *Vibrio* to have E protein which is also found in *Fischeri*, *Enterovibrio* and *Phosphoreum* species. *V. vulnificus* and *V. mimicus* are the only representatives of the Vulnificus and Cholerae clades, respectively, sharing the *eps* cluster. Nevertheless, *V. mimicus* seems to be an atypical species of Cholerae clade [36, 37]. All the 11 Splendidus clade species possess the *eps* cluster. Moreover, all studied species of seven clades examined (Coralliilyticus, Enterovibrio, Fischeri, Mediterranei, Nigripulchritudo, Scophthalmi, Splendidus) share the *eps* cluster. On the other hand, betaine ABC transporter genes were found only in the Splendidus clade with the exception of *V. kanaloae* and *V. toranzoniae.*

Blast search on the NCBI genome sequence database [12] excluding all Vibrionaceae species was used to infer the overall occurrence of the *eps* cluster. Only a few genomes contained homologs to the *eps* genes (Additional file 5). However, in these strains, the gene order is different, some gene blocks are inverted and differentially located on (+) and (−) DNA strands. In addition, several deletions/insertions are observed. The *eps* cluster examined in this paper thus appears as specific to Vibrionaceae and has likely been acquired by horizontal gene transfer in the few other bacteria sharing it.

## Limitations

This identification of a large number of orthologous *eps* clusters within the Vibrionaceae is interesting to obtain EPS glycosaminoglycan-like molecules with distinct structural features. But it necessitates further studies by isolating and characterizing the putative EPS produced to gain insight into the EPS structural features. This is a challenge because EPS production conditions and regulation mechanisms are not fully understood. The characterization of a large number of EPS molecules together with the biosynthesis gene cluster structure would provide a relevant basis to identify the genetic mechanisms of the biosynthesis and open the field of synthetic biology to produce glycosaminoglycan-mimetics.

## Additional files

**Additional file 1.** List of strains studied. The assembly reference is indicated as well as clades.

**Additional file 2.** List of strains and sequence accession numbers used for the MLSA (multilocus sequence analysis).

**Additional file 3.** Proteins encoded by *eps* orthologous clusters in Vibrionaceae. Lines in green (*E. coralii*, *V. breoganii* FF50 and *V. ichthyoenteri*): clusters with specific genes as indicated, Lines in red (*V. ishigakensis*, *V. maritimus*, *V. ponticus*): strains with replicated genes. Locus tags annotated as pseudogene are also identified.

**Additional file 4.** Biodiversity of the *eps* cluster among Vibrionaceae.

**Additional file 5.** Gene clusters retrieved from the NCBI genome database excluding Vibrionaceae that show homology to *eps* cluster.

## Abbreviations

EPS: exopolysaccharide
HA: hyaluronan
MLSA: multilocus sequence analysis
PABC: ABC type phosphate/phosphonate transport system
