# Supplementary material for: Comparative genomics reveals a widespread distribution of an exopolysaccharide biosynthesis gene cluster among Vibrionaceae
Source: BMC Res Notes. 2018 Feb 6;11:102. doi: 10.1186/s13104-018-3214-z (PMC5801674; doi:10.1186/s13104-018-3214-z)
Supplement: Supplementary file 4 — Additional file 4. Biodiversity of the eps cluster among Vibrionaceae. [file 13104_2018_3214_MOESM4_ESM.docx]

| **Strain** |  |
| --- | --- |
| *Aliivibrio fischeri* ES1148 | 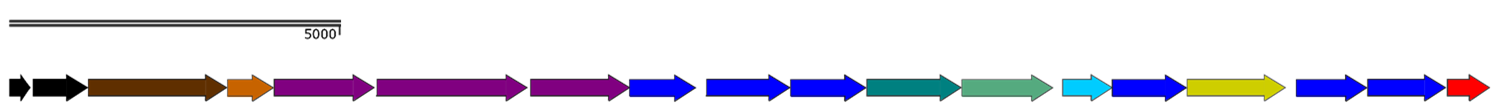 |
| *Aliivibrio fischeri* MJ11 | 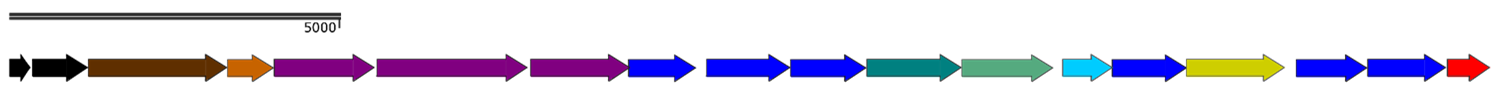 |
| *Aliivibrio logei* | 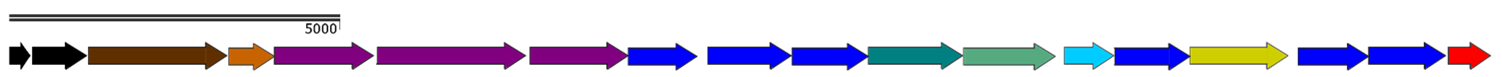 |
| *Aliivibrio salmonicida* | 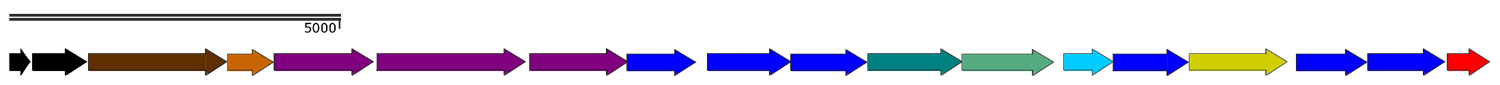 |
| *Aliivibrio wodanis* | 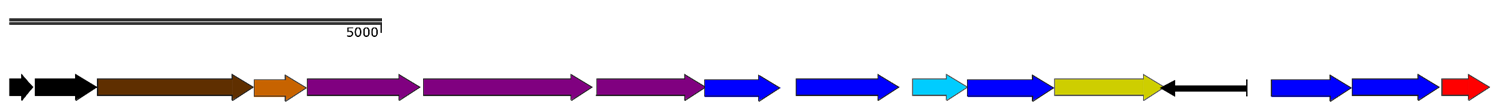 |
| *Enterovibrio calviensis* | 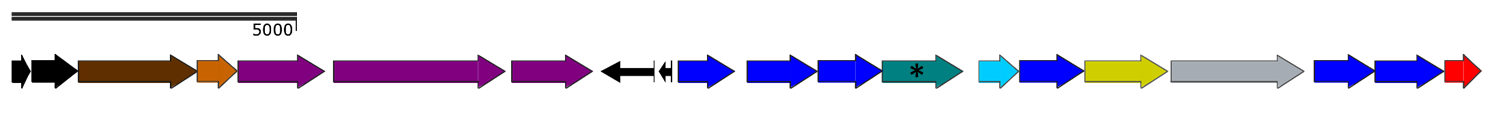 |
| *Enterovibrio coralii* | 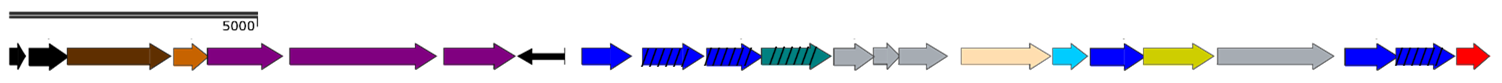 |
| *Photobacterium phosphoreum* | 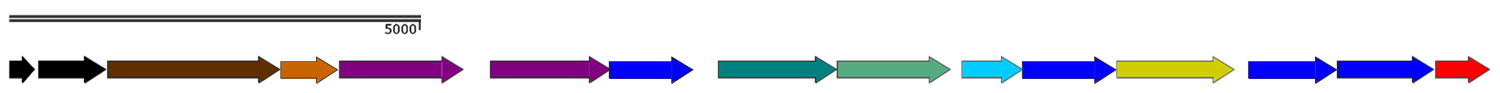 |
| *Vibrio alginolyticus* 12G01 | 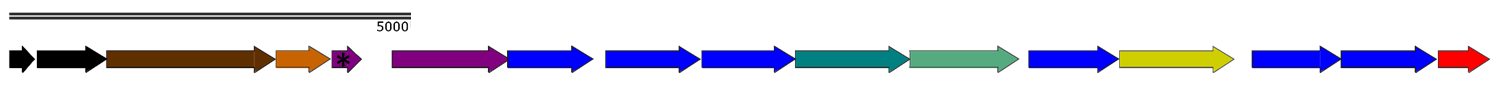 |
| *Vibrio alginolyticus* ATCC17749 | 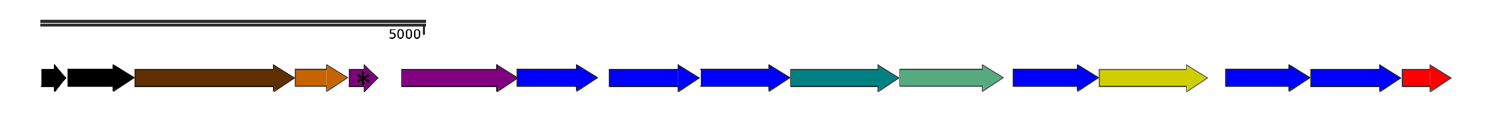 |
| *Vibrio alginolyticus ATCC33787* | 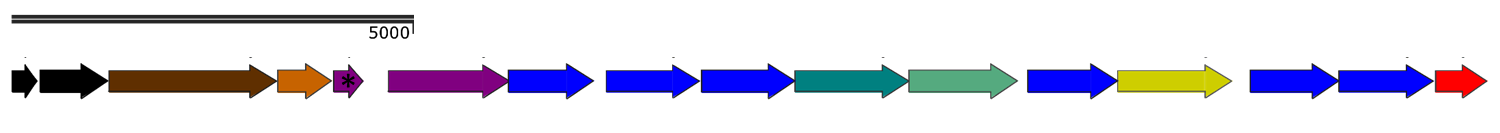 |
| *Vibrio alginolyticus ZJ-T* | 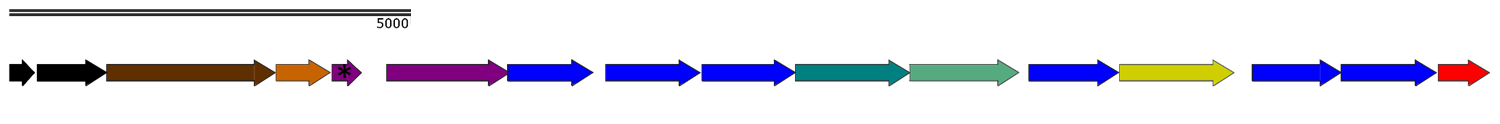 |
| *Vibrio antiquarius Ex25* | 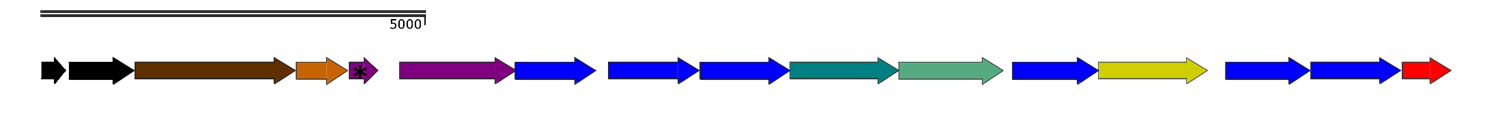 |
| *Vibrio atlanticus* CECT7223 | 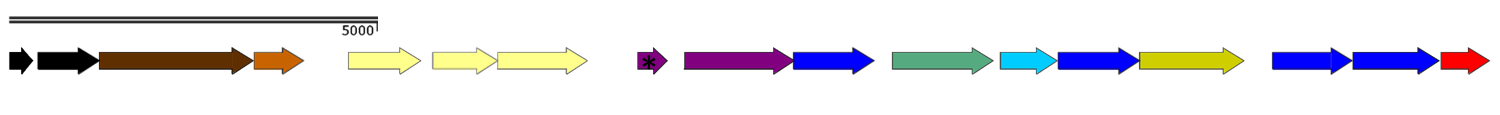 |
| *Vibrio azureus* NBRC104587 | 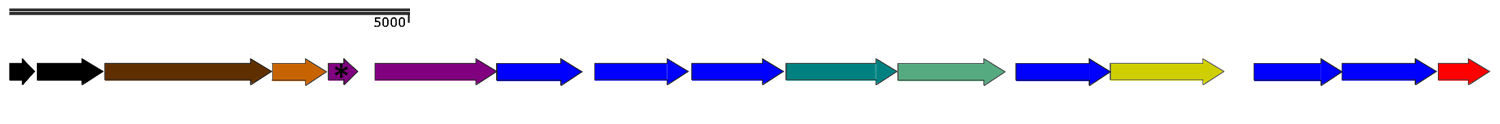 |
| *Vibrio barjaei* | 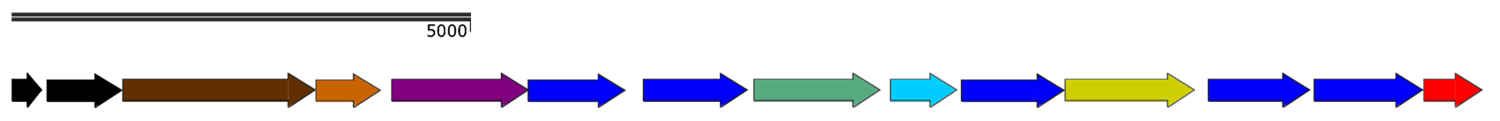 |
| *Vibrio bivalvicida* | 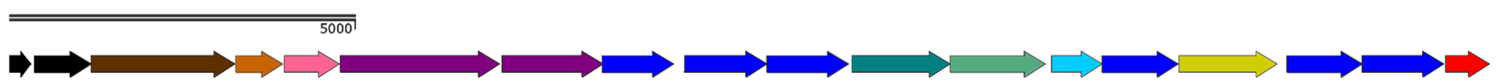 |
| *Vibrio brasiliensis* LMG20546 | 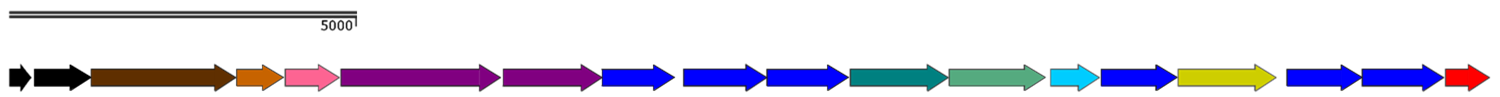 |
| *Vibrio breoganii* FF50 | 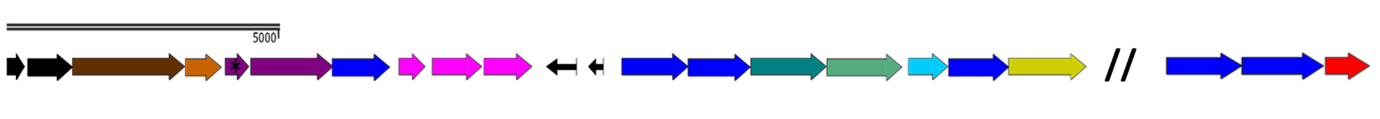 |
| *Vibrio campbellii* BAA1116 | 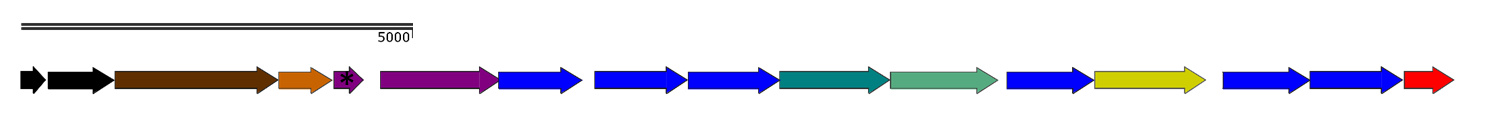 |
| *Vibrio campbellii* NRBC15631 | 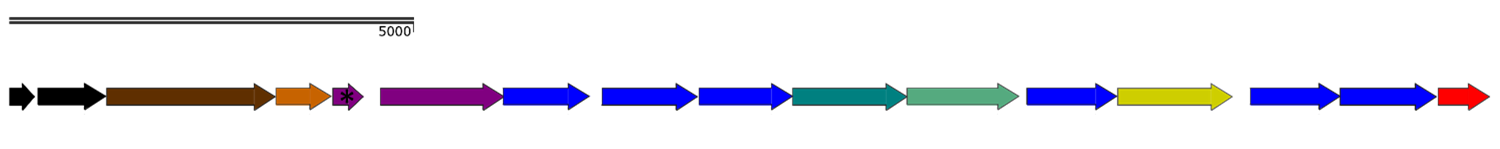 |
| *Vibrio celticus* | 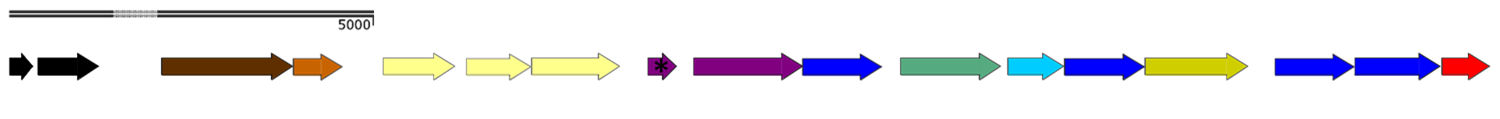 |
| *Vibrio coralliilyticus* | 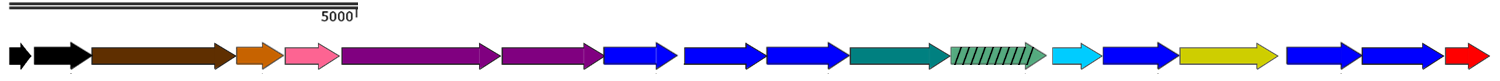 |
| *Vibrio crassostreae* | 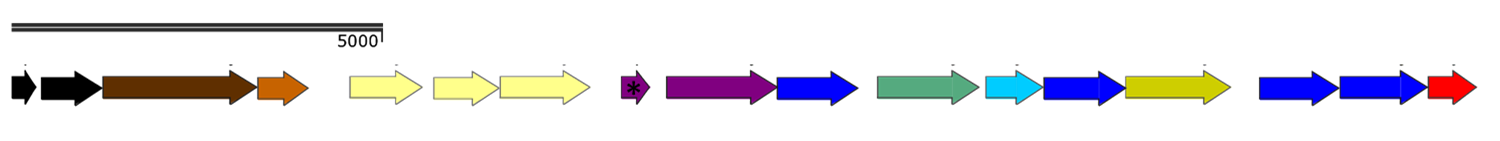 |
| *Vibrio cyclitrophicus* | 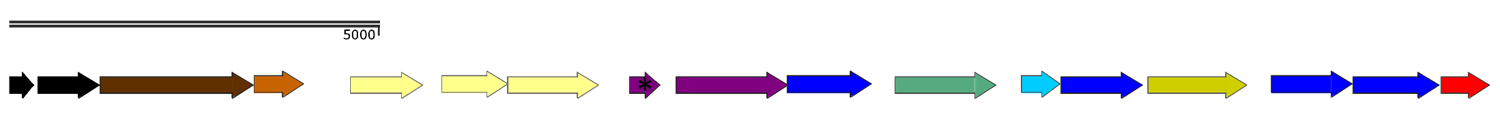 |
| *Vibrio diabolicus* | 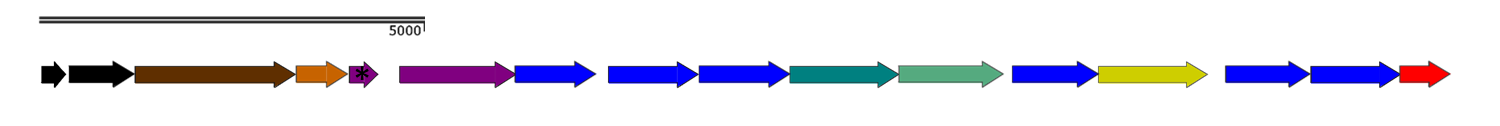 |
| *Vibrio fortis* | 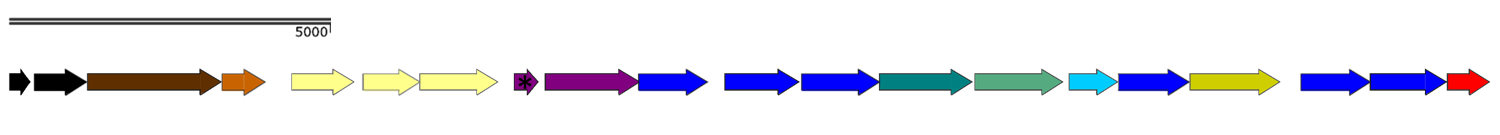 |
| *Vibrio halioticoli* | 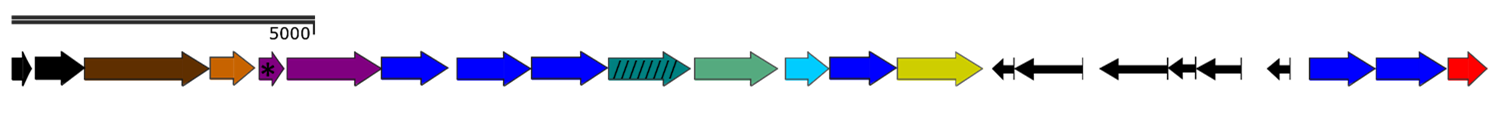 |
| *Vibrio harveyi* ATCC33843 | 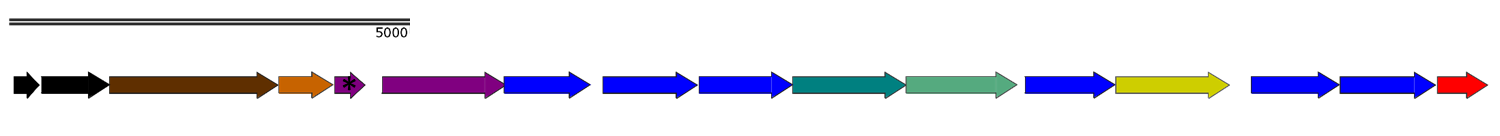 |
| *Vibrio harveyi* ATCC43516 | 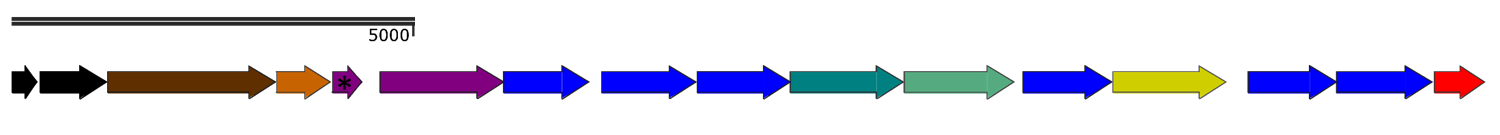 |
| *Vibrio hemicentroti* | 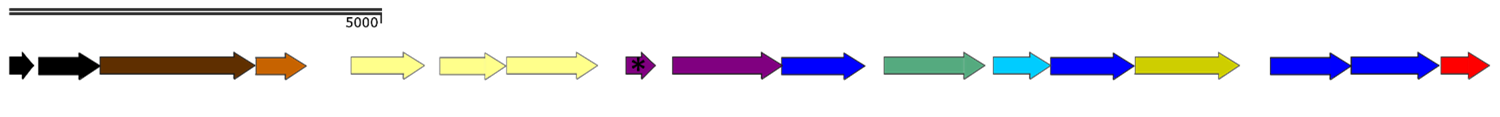 |
| *Vibrio hyugaensis* | 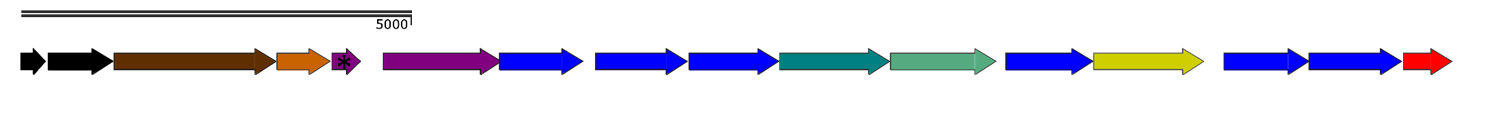 |
| *Vibrio inhibens* | 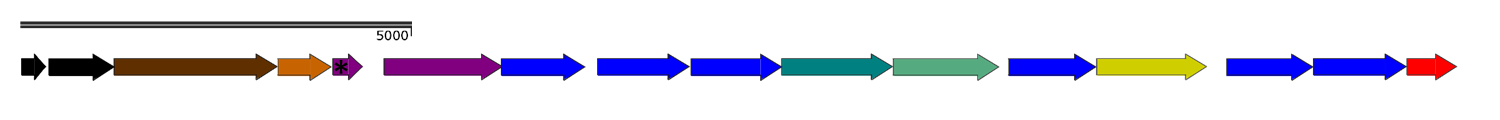 |
| *Vibrio ishigakensis* | 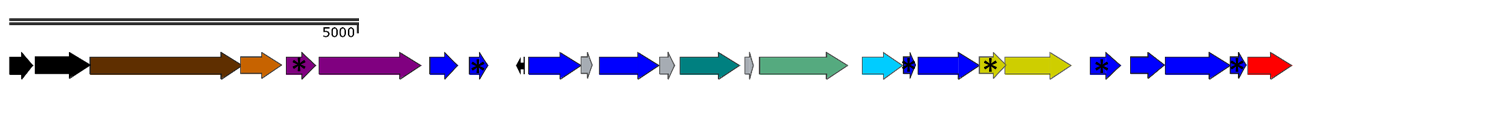 |
| *Vibrio jasicida* | 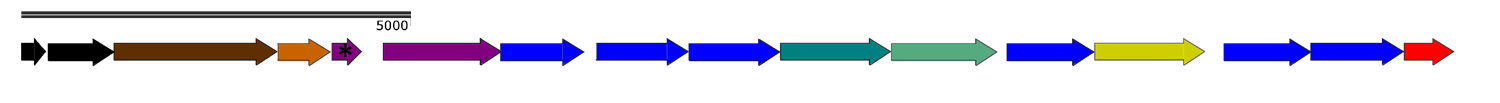 |
| *Vibrio kanaloae* | 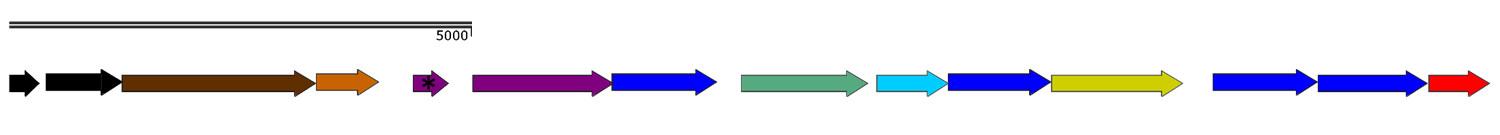 |
| *Vibrio lentus* | 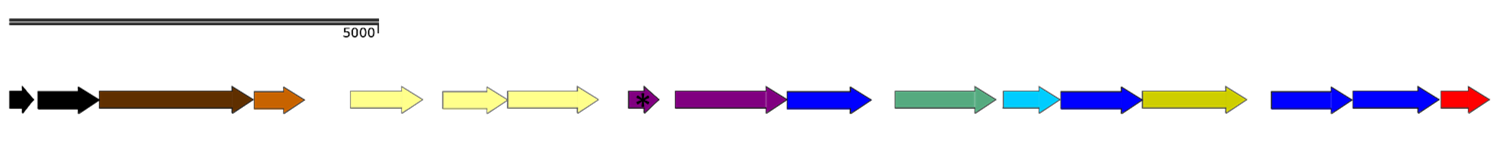 |
| *Vibrio maritimus* | 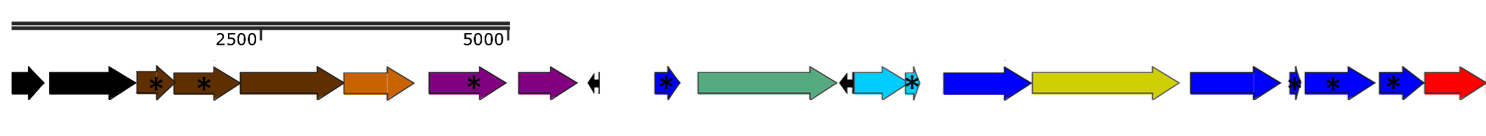 |
| *Vibrio mediterranei* | 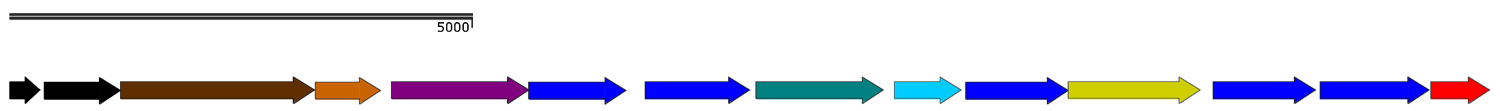 |
| *Vibrio mimicus A*TCC33654 | 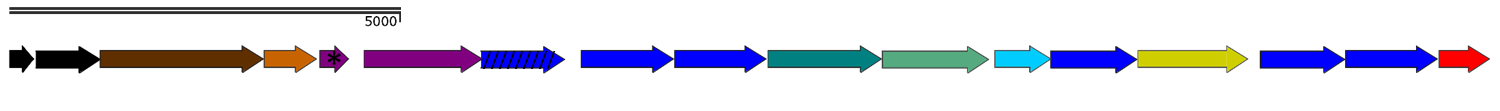 |
| *Vibrio mimicus* SCCF01 | 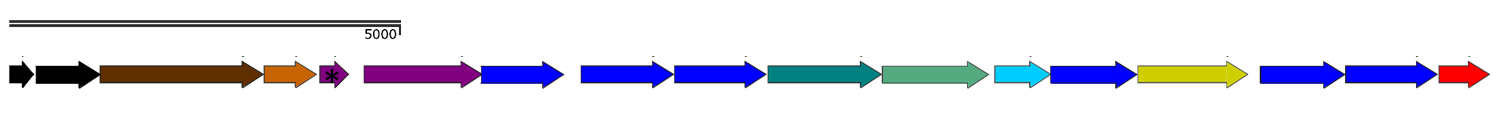 |
| *Vibrio natriegens* ATCC14048 | 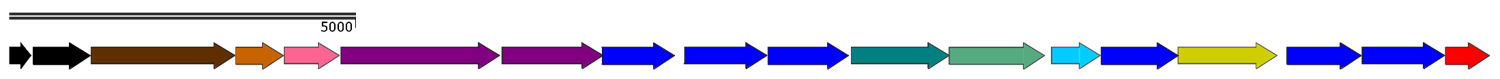 |
| *Vibrio natriegens* CCUG16371 | 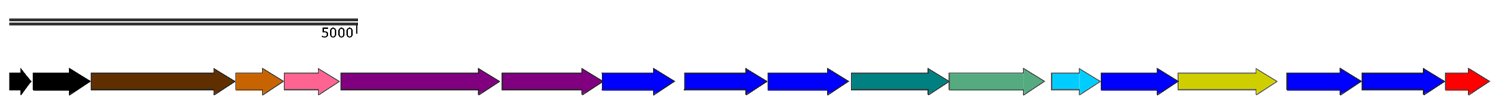 |
| *Vibrio natriegens* CCUG16373 | 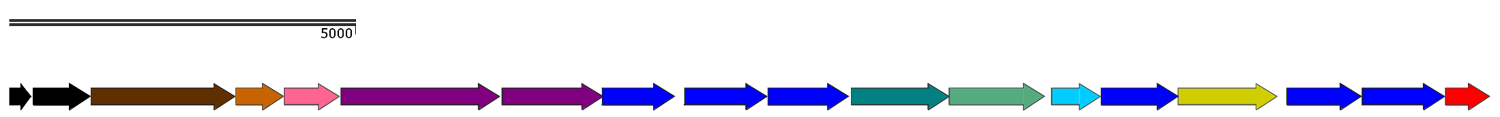 |
| *Vibrio natriegens* CCUG16374 | 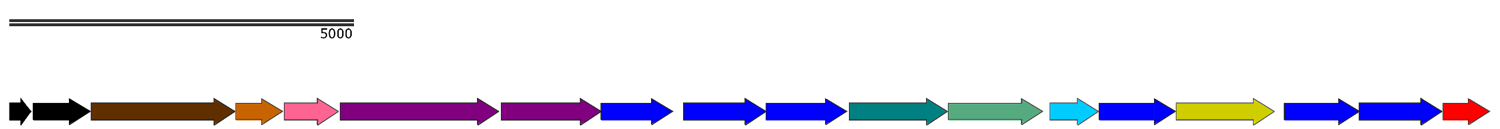 |
| *Vibrio neptunius* | 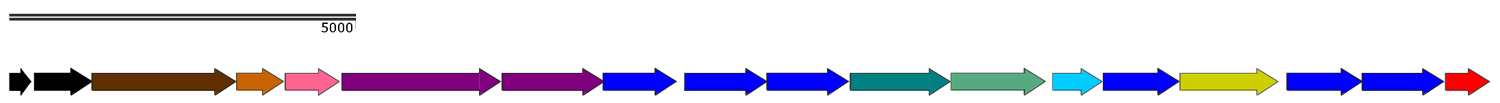 |
| *Vibrio nigripulchritudo* | 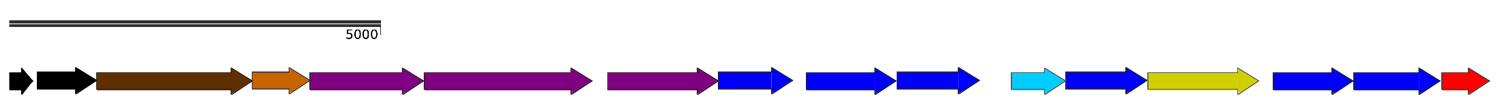 |
| *Vibrio ordalii* ATCC33509 | 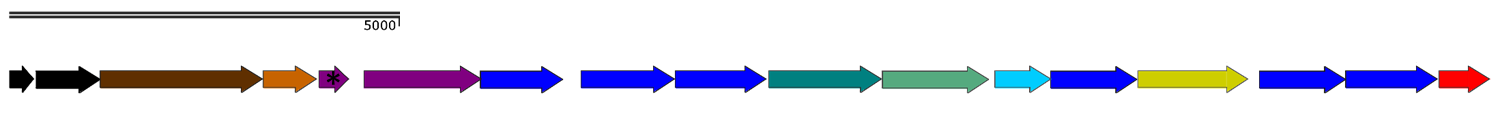 |
| *Vibrio orientalis* | 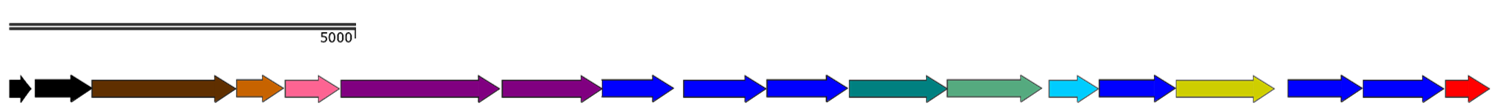 |
| *Vibrio owensii* | 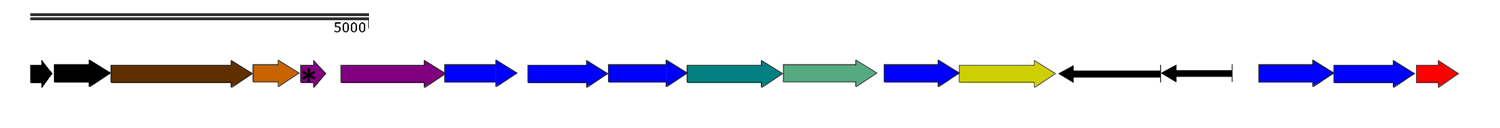 |
| *Vibrio pacinii* | 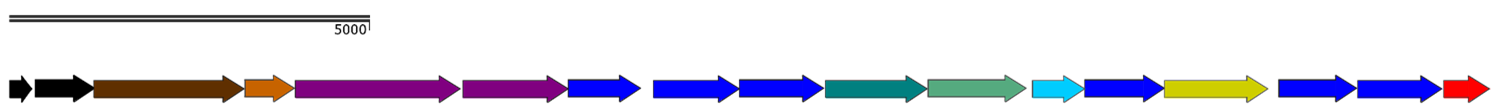 |
| *Vibrio parahaemolyticus* ATCC17802 | 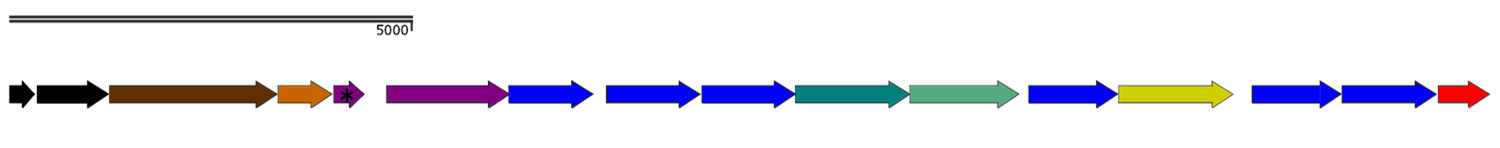 |
| *Vibrio parahaemolyticus* RIMD2210633 | 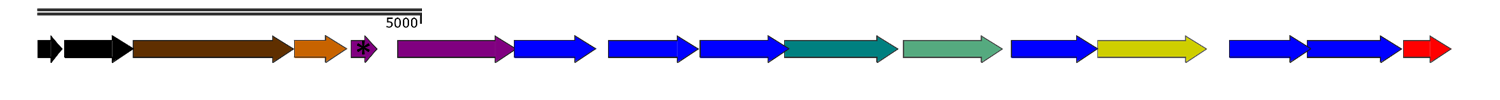 |
| *Vibrio ponticus* | 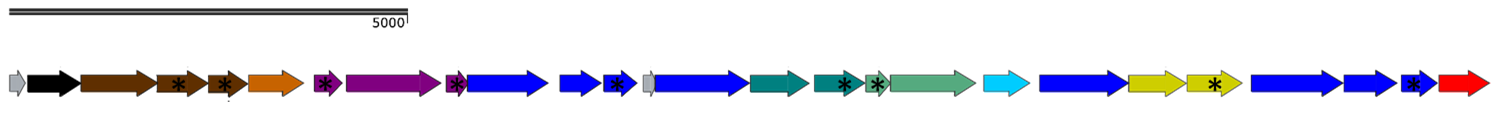 |
| *Vibrio proteolyticus* | 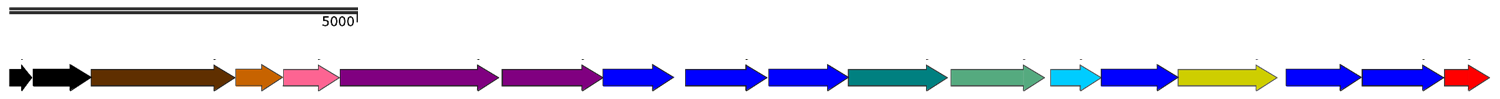 |
| *Vibrio renipiscarius* | 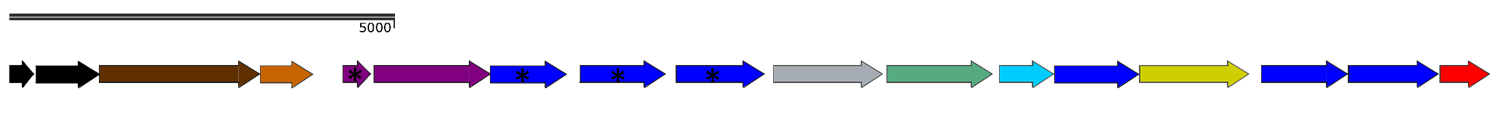 |
| *Vibrio rotiferianus* | 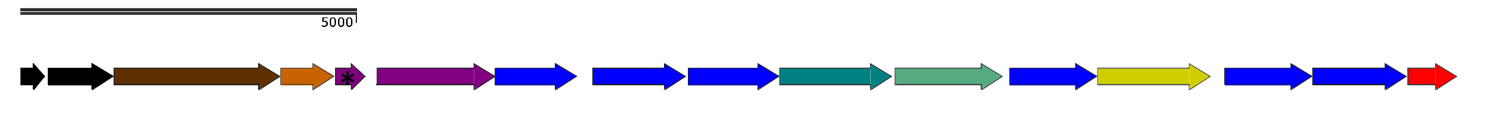 |
| *Vibrio sagamiensis* | 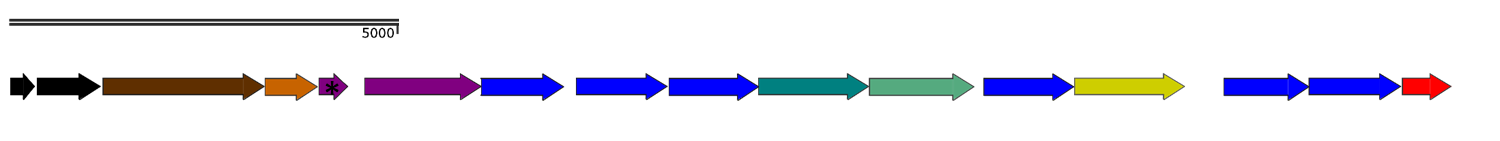 |
| *Vibrio scophthalmi* VS_05 | 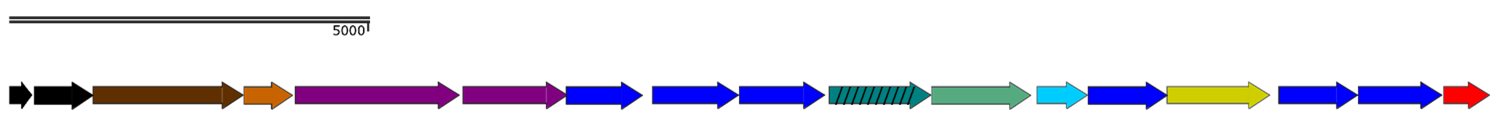 |
| *Vibrio scophthalmi* VS_12 | 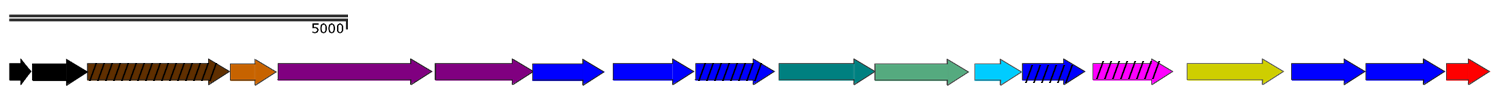 |
| *Vibrio shilonii* | 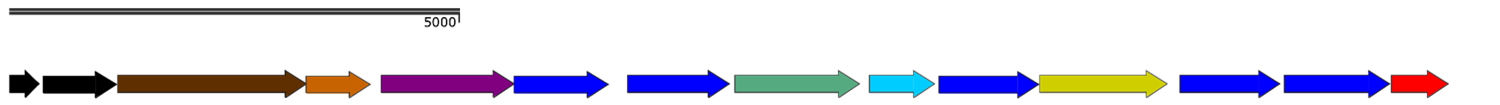 |
| *Vibrio sinaloensis* | 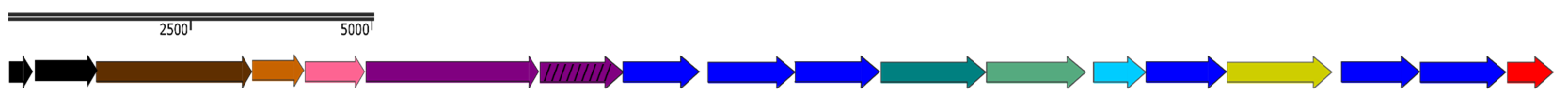 |
| *Vibrio sp. EJY3* | 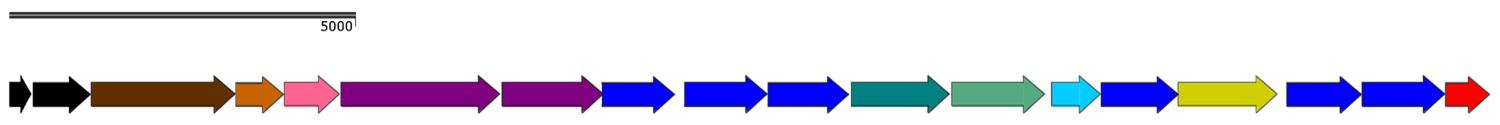 |
| *Vibrio splendidus* | 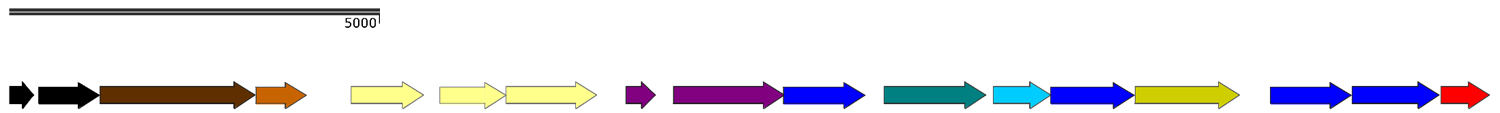 |
| *Vibrio tasmaniensis* | 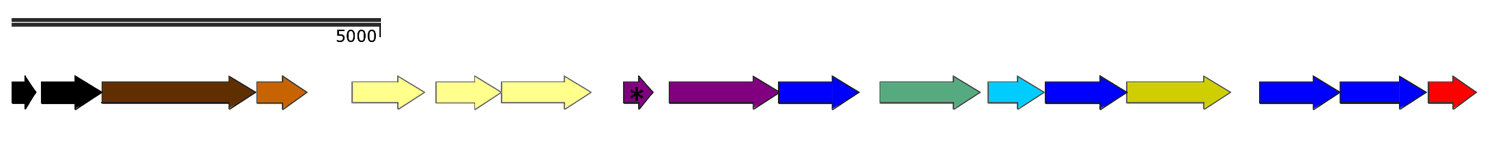 |
| *Vibrio toranzoniae* | 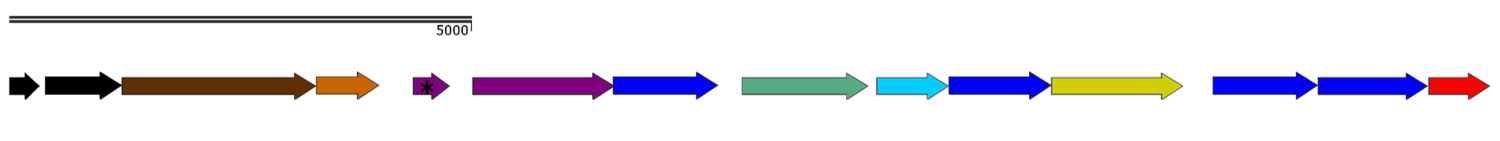 |
| *Vibrio tubiashii* | 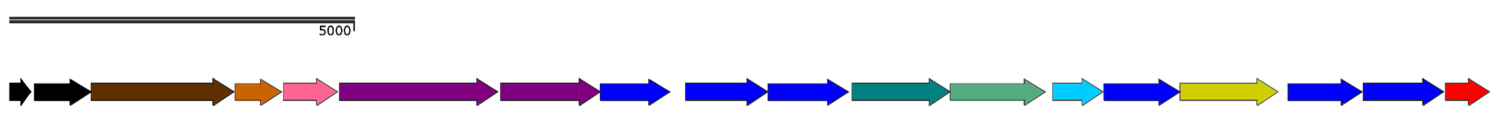 |
| *Vibrio variabilis* | 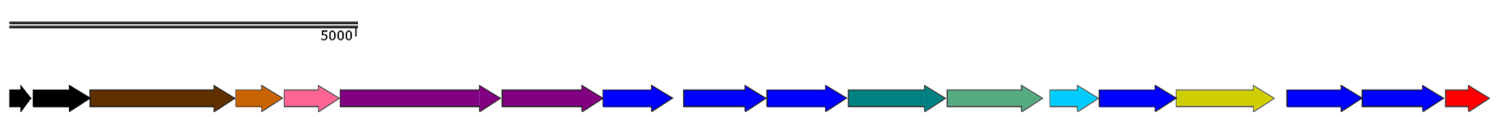 |
| *Vibrio vulnificus* CMCP6 | 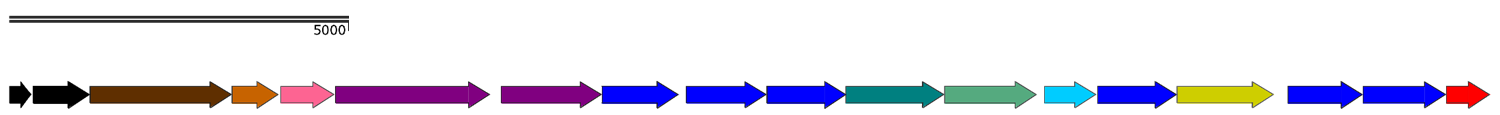 |
| *Vibrio vulnificus* YJ016 | 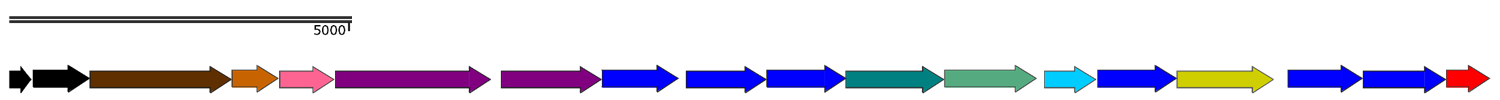 |
| *Vibrio xuii* | 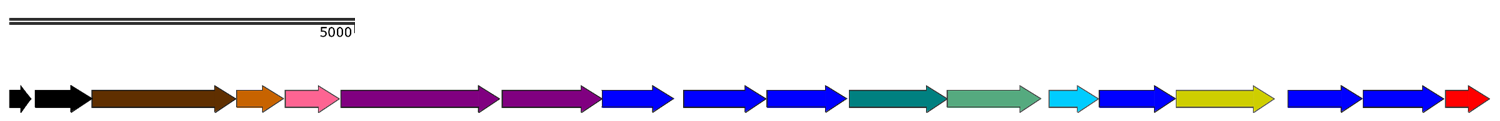 |

**Additional file 4:** Biodiversity of the *eps* cluster among Vibrionaceae. Each arrow represents a gene in the He800-like cluster. Genes are colored on the basis of their predicted function as following: A (■), B (■), C (■), D (■), E (■), F (■), G (■), H (■), I (■), J (■), K (■), L(■), M (■), N (■), O (■), P (■), Q (■), R (■), PABC (■), X2 (■), X3 (■), X4 (■),Transposase–integrase (■),Glutamine-fructose-6-phosphate aminotransferase (■), Hypothetical protein (■) (DOCX, 1725 KB).
